# *TASK-3* Gene Knockdown Dampens Invasion and Migration and Promotes Apoptosis in KATO III and MKN-45 Human Gastric Adenocarcinoma Cell Lines

**DOI:** 10.3390/ijms20236077

**Published:** 2019-12-02

**Authors:** Rocio Cikutović-Molina, Andres A. Herrada, Wendy González, Nelson Brown, Leandro Zúñiga

**Affiliations:** 1 Center for Medical Research, School of Medicine, Universidad de Talca, Talca 3460000, Chile; rcikutovic@gmail.com (R.C.-M.); nbrown@utalca.cl (N.B.); 2 Programa de Investigación Asociativa en Cáncer Gástrico (PIA-CG), Universidad de Talca, Talca 3460000, Chile; 3 Lymphatic and Inflammation Research Laboratory, Faculty of Health Sciences, Institute of Biomedical Science, Universidad Autónoma de Chile, Talca 3467987, Chile; andres.herrada@uautonoma.cl; 4 Center for Bioinformatics and Molecular Simulations (CBSM), Universidad de Talca, Talca 3460000, Chile; wgonzalez@utalca.cl; 5 Millennium Nucleus of Ion Channels-Associated Diseases (MiNICAD), Universidad de Talca, Talca 3460000, Chile

**Keywords:** TASK-3, gastric cancer, cell proliferation, apoptosis, migration, invasion

## Abstract

Incidence and mortality of gastric cancer is increasing worldwide, in part, because of the lack of new therapeutic targets to treat this disease. Different types of ion channels participate in the hallmarks of cancer. In this context, ion channels are known to exert control over the cell cycle, mechanisms that support survival, angiogenesis, migration, and cell invasion. In particular, TASK-3 (*KCNK9*), a member of the K2P potassium channel family, has attracted much interest because of its oncogenic properties. However, despite multiple lines of evidence linking TASK-3 to tumorigenesis in various types of cancer, its relationship with gastric cancer has not been fully examined. Therefore, we set out to assess the effect of TASK-3 gene knockdown on KATO III and MKN-45 human gastric adenocarcinoma cell lines by using a short hairpin RNA (shRNA)-mediated knockdown. Our results demonstrate that knocking down TASK-3 reduces cell proliferation and viability because of an increase in apoptosis without an apparent effect on cell cycle checkpoints. In addition, cell migration and invasion are reduced after knocking down TASK-3 in these cell lines. The present study highlights TASK-3 as a key protein involved in migration and cell survival in gastric cancer and corroborates its potential as a therapeutic target for gastric cancer treatment.

## 1. Introduction

Under physiological conditions, cells in a multicellular organism proliferate in a harmonious and orderly way, driven by a series of complex mechanisms involving intercellular communication [1]. Thus, the balance between growth and death contributes to the maintenance of the structure of organs and tissues [2]. Not surprisingly, alterations in genes that regulate these processes can lead to cancer [3]. Cancer initiation and progression involves a series of genetic and epigenetic alterations that render cells able to proliferate without control and to invade and colonize distant organs and tissues [4]. The accumulation of successive gene mutations promotes the acquisition of the “hallmarks of cancer”, which include an increase in cell proliferation [5], resistance to apoptosis [6], metabolic changes [7], genetic instability [8], induction of angiogenesis [9], and an increase in the migratory capacities of cells [10]. Understanding these processes is key to prevention, effective diagnosis, and the design of new therapeutic strategies [11].

Gastric cancer is the sixth most frequent type of cancer and the third cause of cancer-related deaths worldwide [12]. The treatment of gastric cancer is fundamentally surgical, with current protocols including adjuvant chemotherapy, radio-chemotherapy, or monoclonal antibodies [13,14]. However, these treatments are not suitable for all patients, and new therapeutic strategies still need to be developed [15].

In last 10 years, the role of ion channels in the pathogenesis of various diseases [16] has been demonstrated. Diseases involving dysregulation of channels include migraines [17], cardiac arrhythmias [18], ulcerative colitis [19], Alzheimer’s [20], and cancer [21]. Regarding cancer, different types of ion channels are known to participate in the orchestration of different hallmarks of cancer [22], in the control of the cell cycle [23], in the mechanisms responsible for survival and apoptosis [24], as well as in cell migration and invasion [25].

TASK-3 (*KCNK9* or K2P9.1) is a potassium channel from the K_2P_ family with the ability to form functional homo- or heterodimers [26]. TASK-3 expression is low in most normal tissues, except the brain where it is mainly found in neurons of the central nervous system, contributing to the maintenance of the resting membrane potential and control of the action potential [27]. Interestingly, overexpression of TASK-3 in various tumors has been reported [28,29,30,31,32]. In particular, overexpression of TASK-3 has been observed in 90%, 44%, and 35% of ovarian [28], breast [29], and lung cancers [32], respectively. Additionally, a lower percentage of TASK-3 overexpression in colorectal cancer [30] and melanomas [31] has been reported.

Several research groups have assessed the oncogenic properties of TASK-3 in vitro. TASK-3 has been involved in mechanisms of apoptosis evasion [33] that favor the survival of cells under stress conditions, such as serum deprivation or hypoxia [29]. Thus, Mu et al. [29] observed that the overexpression of TASK-3 in cells (C8) with low tumorigenicity leads to the acquisition of resistance to cell death and enhanced tumorigenesis. So far, however, there is no clear evidence as to how TASK-3 might contribute to these processes at the molecular level. One hypothesis suggests that the control of K^+^ ions and water movement could play a role [34,35]. Also, a recent study showed that knocking down TASK-3 in breast cancer cells resulted in the induction of cellular senescence and cell cycle arrest [36]. Likewise, it was demonstrated that the use of a dominant-negative form of TASK-3 (TASK-3 G95E) [37], or the use of a monoclonal antibody against its extracellular domain [38], led to a decrease in proliferation due to apoptosis induction in lung and breast carcinoma cells, respectively. In both studies, reduced expression or blockade of TASK-3 function led to reduced tumor growth and metastasis in a mouse model, confirming the causal role of this potassium channel on the tumorigenic process [37,38].

In the present work, we evaluated the expression of TASK-3 in KATO III and MKN-45 human gastric carcinoma cells. In addition, the effects of knocking down TASK-3 on the ability of these cells to proliferate, migrate, and invade are described. Our results demonstrate that while knocking down TASK-3 induces apoptosis in a percentage of cells, surviving cells remain defective in migration and invasion.

## 2. Results

### 2.1. Expression and Knockdown of TASK-3 in KATO III and MKM-45 Cell Lines

**Figure 1:**
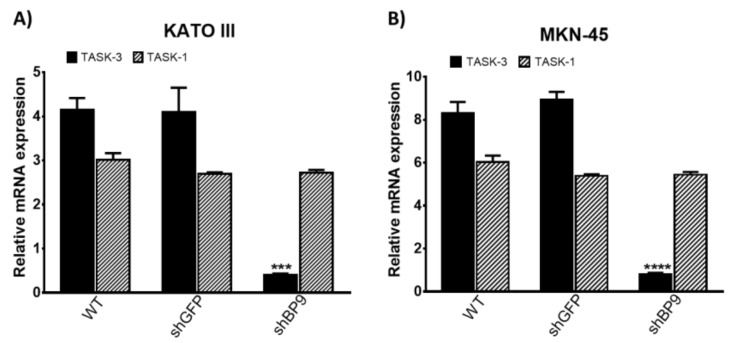
mRNA expression of TASK channels in KATO III and MKN-45 cell lines. (**A**,**B**) expression of TASK-3 (*KCNK9*) and TASK-1 (*KCNK3*) in wild-type (WT) cells or cells transduced with shRNAs directed against GFP (shGFP) or TASK-3 (shBP9). Gene expression was normalized against the expression of the gene encoding for the ribosomal protein L19 (RPL19) using the ΔΔ*C*t method. Error bars correspond to mean ± SEM (*n* = 3). *** *p* < 0.001, **** *p* < 0.0001, compared with WT, based on ANOVA followed by Dunnett’s test.

Two human gastric adenocarcinoma cell lines, KATO III and MKN-45, were used throughout this work. We first set out to detect the mRNA and protein levels of TASK-3 and the highly homologous TASK-1 channel. Of note, TASK-1 is known to be able to form heterodimers with TASK-3 [26]. As shown in Figure 1, mRNA transcripts for TASK-3 and TASK-1 genes were detectable in KATO III (Figure 1A) and MKN-45 (Figure 1B) cells. There were no differences in the mRNA levels of TASK-1 and TASK-3 between cells not transduced and cells that were transduced with the shGFP control. In contrast, cells transduced with the shRNA targeting TASK-3 (shBP9) showed a significant reduction in the mRNA levels of TASK-3. These results indicate an efficient TASK-3 downregulation in both cell lines. Unlike TASK-3, the mRNA levels of TASK-1 did not show a statistically significant reduction in cells transduced with shBP9, attesting for the specificity of the short hairpin used and ruling out compensatory changes in the expression of TASK-1.

**Figure 2:**
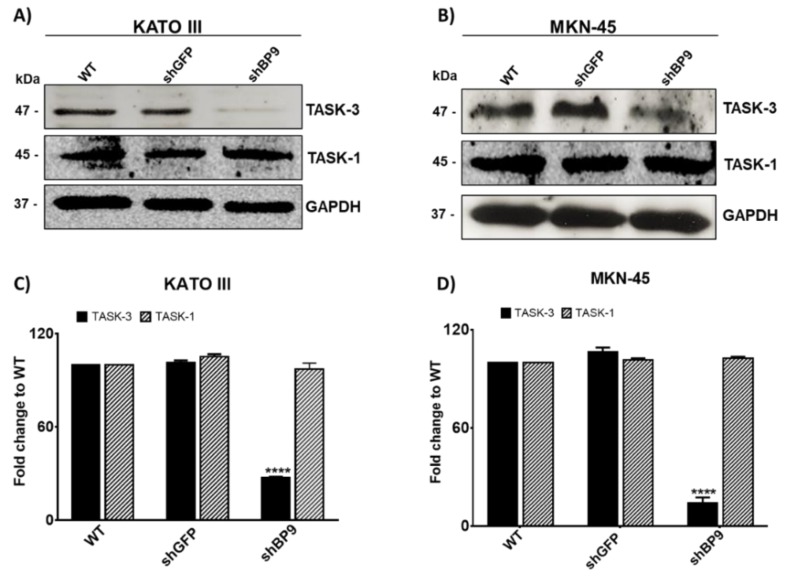
Protein levels of TASK-3 and TASK-1 in KATO III and MKN-45 cell lines. (**A**,**B**) Representative immunoblots for TASK-3, TASK-1, and GAPDH are shown for wild-type (WT) cells as well as cells transduced with shRNAs against GFP (shGFP) or TASK-3 (shBP9). (**C**,**D**) Relative abundance of TASK-3 and TASK-1 protein based on densitometric analyses. Data are expressed as mean ± SEM of three independent experiments. **** *p* < 0.0001, compared with WT, based on ANOVA followed by Dunnett’s test.

We next analyzed the protein levels of TASK channels by western blotting (Figure 2). We confirmed the presence of both channels in KATO III (Figure 2A) and MKN-45 (Figure 2B) cells, indicating that these cells not only generated the relevant transcripts but also processed them in order to generate protein. As shown in Figure 2C,D. TASK-3 protein levels were reduced in both cell lines after being transduced with shBP9, corroborating the effectivity of the shRNA-mediated knockdown of TASK-3. As expected, TASK-1 levels did not change, indicating that the short hairpin used was TASK-3-specific. In addition, no compensatory variations in the protein levels of TASK-1 were observed in TASK-3-depleted cells. 

### 2.2. TASK-3 Knockdown Inhibits Cell Proliferation and Viability in KATO III and MKN-45 Cells

**Figure 3:**
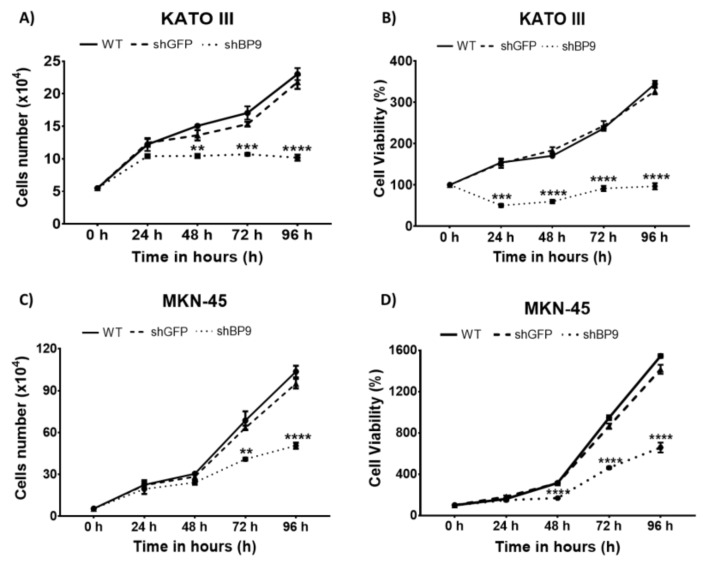
Effect of knocking down TASK-3 on cell proliferation and viability. (**A**,**C**) Proliferation curves of wild-type (WT) KATO III and MKN-45 cells, or the same cells transduced with shRNAs against GFP (shGFP) or TASK-3 (shBP9) after 0, 24, 48, 72, and 96 h of incubation. (**B**,**D**) Cell viability curves as a percentage of viability of KATO III and MKN-45 cells under the indicated conditions following an incubation of 0, 24, 48, 72, and 96 h. Error bars represent the mean ± SEM of three independent experiments. ** *p* < 0.01, *** *p* < 0.001, **** *p* < 0.0001, compared with WT based on ANOVA followed by Tukey’s post-test.

We next investigated the effects of TASK-3 depletion in cell proliferation and viability. Both characteristics were determined in parallel, generating a curve from 0 to 96 hours (h). Figure 3A shows the proliferation curves for KATO III cells under different experimental conditions. As expected, there were no significant differences in the kinetics of proliferation between the wild-type (WT) and shGFP conditions. In contrast, cells transduced with shBP9 showed a significant decrease (*p* < 0.01) in proliferation after 48 h, indicating that TASK-3 was necessary to drive proliferation in these cells. Similar findings were observed in MKN-45 cells (Figure 3C).

Next, analyses of cell viability were carried out. The results are shown in Figure 3B,D. As expected, WT KATO III cells (Figure 3B), and the same cells transduced with shGFP, did not show significant differences in their viability curves. In contrast, shBP9-tranduced KATO III cells showed a significant reduction (*p* < 0.001) in cell viability that was evident after 24 h of incubation. The same response was observed in MKN-45 cells, although the reduction in cell viability was evident after 48 h of incubation (Figure 3D). This result suggests that knocking down TASK-3 not only altered cell proliferation but also exerted a cytotoxic role.

Of note, knocking down TASK-3 in both cell lines led to a significant reduction in cell viability (Figure 3B,D) that was evident 24 h earlier than the decrease in cell proliferation (Figure 3A,C). For example, KATO III cells showed a significant decrease in cell proliferation beginning 48 h after incubation, while the viability of these cells showed a significant reduction 24 h earlier. Similarly, MKN-45 cells showed a significant decrease in cell proliferation beginning 72 h after incubation, while the viability of these cells was evident already 48 h after incubation. This difference could be explained by the fact that in cell proliferation assays, the total number of cells for each condition was quantified, while for cell viability assays only those cells with intact membranes were quantified. Thus, it is probable that a fraction of the total population of cells was undergoing an early apoptotic process or another phenomenon that involves the beginning of an alteration at least at the membrane level.

### 2.3. TASK-3 Knockdown in KATO III and MKN-45 Cells Triggers an Increase in Apoptosis without Altering the Cell Cycle

**Figure 4:**
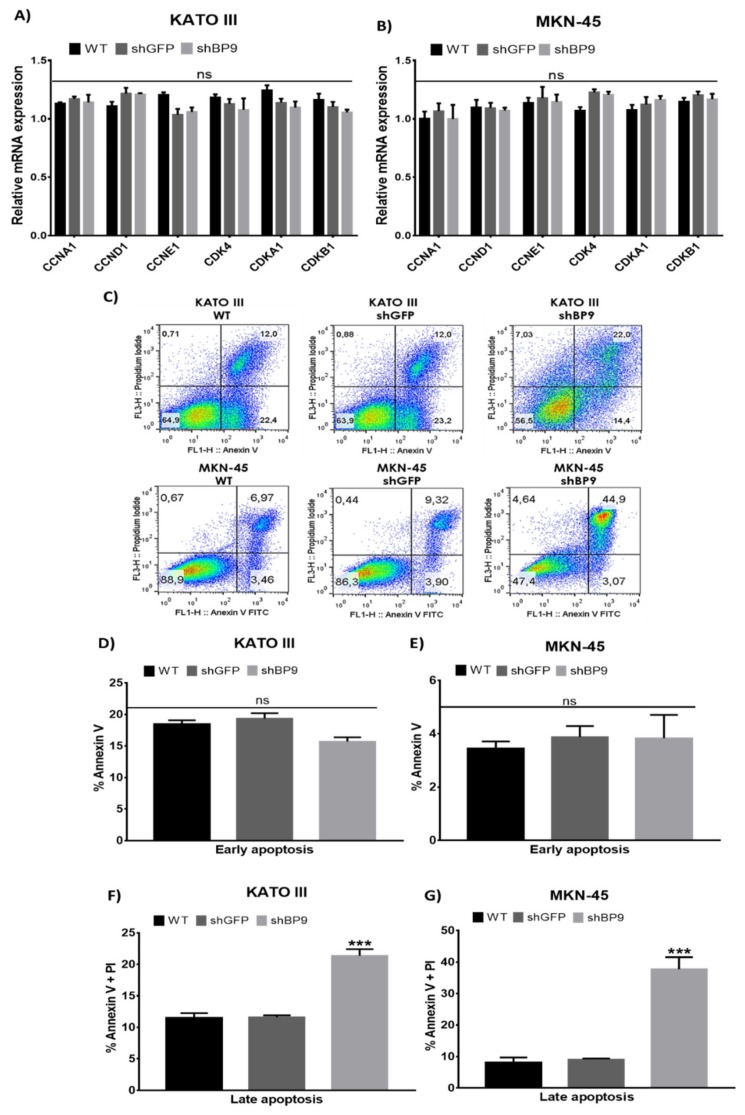
Effects of knocking down TASK-3 on cell cycle regulators and apoptosis. (**A**,**B**) The expression of cell cycle regulators in wild-type (WT) KATO III and MKN-45 cells, or the same cells transduced with an shRNA against GFP (shGFP) or TASK-3 (shBP9). (**C**) Cytometry analyses of apoptosis using annexin V and propidium iodide (PI). (**D**–**G**) Percentage of cells in early or late apoptosis based on percentages obtained in C. Error bars correspond to the mean ± SEM of three independent experiments. Cell cycle markers were evaluated by a two-way ANOVA test with a *p* ≤ 0.05. Early and late apoptosis were evaluated by ANOVA followed by Dunnett’s test. *** p <0.001, compared to WT. ns = no significant difference.

To further understand the cellular mechanisms involved in the reduced cell proliferation and viability observed in TASK-3-deficient cells, cell cycle and apoptosis were evaluated. Figure 4A,B shows the results of mRNA expression, evaluated by qRT-PCR, for different cell cycle regulators involved in the G1/S transition of the cell cycle (*CCNA1*, *CCND1,* and *CCNE1*, encoding cyclins A1, D1, and E1, respectively; *CDK4*, encoding cyclin-dependent kinase 4; and *CDKN1A* and *CDKN1B*, encoding cyclin-dependent kinase inhibitors 1A and 1B, also known as p21 and p27, respectively) [39]. For both KATO III (Figure 4A) and MKN-45 (Figure 4B) cells, the expression levels of cell cycle regulators did not show any significant differences. In accordance with these results, flow cytometry based cell cycle analyses of MKN-45 cells, subjected to different experimental conditions, failed to reveal any significant differences in the proportions of cells in different phases of the cell cycle (G1, S, and G2/M) (Supplementary Figure S1). These results indicated that changes in cell proliferation and cell viability observed in response to reduced levels of TASK-3 could not be explained by cell cycle arrest.

Based on these results, we next set out to assess apoptosis (Figure 4C–G). Figure 4C shows representative dot plots obtained by flow cytometry after staining KATO III and MKN-45 cells, under different conditions, with annexin V and/or propidium iodide (PI). For a better analysis of these results, Figure 4D,E shows the percentage of cells in early apoptosis (annexin V-positive) and late apoptosis (annexin V- and PI-positive). As shown in Figure 4D, the number of KATO III cells undergoing early apoptosis was not significantly different between all three conditions evaluated. However, a higher proportion of KATO III cells with reduced expression of TASK-3 was detected in late apoptosis (*p* < 0.001) (Figure 4F). These results indicated that TASK-3 depletion could trigger apoptosis in KATO III cells. Likewise, while the number of MKN-45 cells in early apoptosis did not change under different conditions (Figure 4E), a higher proportion of MKN-45 cells in late apoptosis (Figure 4G) was evident following TASK-3 depletion (*p* < 0.001). Thus, similar to KATO III cells, TASK-3 depletion in MKN-45 cells could trigger apoptosis. Altogether, these results indicated that reduced cell proliferation and viability upon depletion of TASK-3 most probably was due to induction of apoptosis.

### 2.4. Inhibition of Cell Migration and Invasion Following TASK-3 Depletion

**Figure 5:**
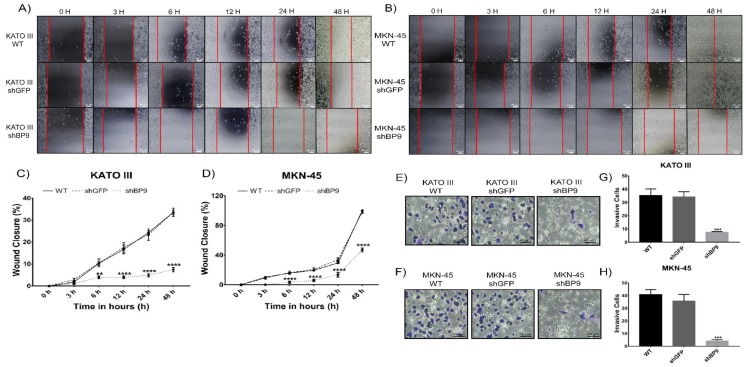
Effect of knocking down TASK-3 on cell migration and invasion in KATO III and MKN-45 cells. (**A**,**B**) Wound healing assays: images obtained at 0, 3, 6, 12, 24, and 48 h after scratch formation. (**C**,**D**) Percentage of wound closure for both cell lines in each condition. Scale bar 40 µm. (**E**,**F**) Transwell invasion assays: representative images obtained for KATO III and MKN-45 wild-type (WT), or cells transduced with shGFP (control) and shBP9 (experimental) cells. Scale bar 40 µm. (**G**−**H**) The number of invasive cells for both cell lines in each condition. Data are shown as mean ± SEM of three independent experiments. Wound closure percentage was assessed by ANOVA followed by Tukey’s post-test. ** *p* < 0.01, **** *p* < 0.0001. Cell invasion was evaluated by ANOVA followed by Dunnett’s test. *** *p* < 0.001, compared to WT.

The role of TASK-3 in the ability of gastric adenocarcinoma cells to migrate and invade was next tested. In order to assess migration, wound healing assays were performed 0, 3, 6, 12, 24, and 48 h after wounding (Figure 5A–D). Panels A and B of Figure 5 show representative images of experiments performed in KATO III and MKN-45 cells, respectively.

As shown in Figure 5C, WT and shGFP-transduced KATO III cells demonstrated similar migratory capabilities. In contrast, knocking down TASK-3 led to a significant decrease (*p* < 0.01) in cell migration that was evident 6 h after wounding. Similarly, while WT and shGFP-transduced MKN-45 cells (Figure 5D) displayed similar cell migration capabilities, shBP9-transduced MKN-45 cells demonstrated a significant decrease (*p* < 0.0001) in cell migration 6 h after wounding. Accordingly, similar results were obtained by transwell assay for both cell lines (data not shown). These results indicate that TASK-3 might play a role in cell migration of gastric adenocarcinoma cells.

In order to assess invasion, transwell assays were performed for both cell lines after knocking down TASK-3 (Figure 5E–H). Panels E and F of Figure 5 show representative images of experiments performed in KATO III and MKN-45 cells, respectively. As expected, WT and shGFP-transduced KATO III cells (Figure 5G) were not significantly different in their ability to invade. In contrast, the total number of invasive cells was significantly diminished (*p* ˂ 0.001) in shBP9-transduced KATO III cells. Similarly, MKN-45 cells showed the same pattern of invasion (Figure 5H). In conjunction, these results indicated that TASK-3 might play a role in the invasive capabilities in gastric adenocarcinoma cells.

## 3. Discussion

To our knowledge, this is the first report that explores the oncogenic role of the potassium channel TASK-3 in gastric cancer cells. While the role of growth-promoting pathways in cancer has been well established [40], the role of ion channels in this disease has only recently been appreciated [41]. This appreciation has also been boosted by the fact that ion channels represent attractive therapeutic targets [41,42].

Our results indicate that TASK-3 is expressed in gastric adenocarcinoma cell lines, showing a 4- to 10-fold amplification at the mRNA level. These results are concordant with the expression levels of the channel that had been previously reported for breast [29], lung [37], ovary [28], melanoma [31], and colon [30] cancers. These results were expected since a greater immune reaction for TASK-3 has been shown in gastric tumor tissues compared to normal tissues [43]. In gastric tumors, TASK-3 immune detection was particularly prominent at the perinuclear membrane, while in normal gastric tissue, both cytoplasmic and perinuclear membrane distributions were evident [43]. In addition, a higher expression of TASK-3 correlates with poor patient survival according to the data provided by The Human Protein Atlas database [43,44]. Nevertheless, the mechanism by which TASK-3 might confer a proliferative advantage to tumor cells is not completely clear.

After confirming TASK-3 overexpression in KATO III and MKN-45 cells, knockdown experiments aimed to reduce its expression in these cells were carried out. For this, a shRNA construct, previously tested and validated, was used [36]. Our results confirmed that the knockdown was successful and specific, since the mRNA and protein levels of the TASK-1 channel were not altered.

Importantly, TASK-3 depletion demonstrated a significant reduction in cell proliferation in both KATO III and MKN-45 cells. Our results are in agreement with the study by Pei et al. [37]. After overexpressing a nonfunctional mutant of TASK-3 in a lung carcinoma cell line, these authors reported ≥50% reduction in proliferation compared to those cells that presented the channel in its native form [37]. In the same study, the authors reported that overexpression of the mutant version of TASK-3 led to less tumor development in vivo [37]. In addition, it has been demonstrated that the TASK-3 inhibitors zinc and methanandamide reduced cell proliferation in ovarian carcinoma cells [28].

In order to determine if the proliferative impairment observed in TASK-3-deficient cells was due to a cytotoxic effect, cell viability was examined by the trypan blue exclusion assay. Our results are concordant with a significant decrease in the percentage of viable cells upon TASK-3 depletion, indicating that reduced proliferation mainly is due to a cytotoxic effect. These results are in agreement with previous work carried out in lung [37], breast [38], and melanoma [45] tumor cell lines. Sun et al. [38] demonstrated that the use of a monoclonal antibody against TASK-3 provokes a significant decrease in cell viability in lung and breast cancer cell lines. On the other hand, TASK-3 knockdown in WM35 and A2058 breast cancer cell lines led to a significant decrease in cell viability resulting from an increase in apoptosis without increasing necrosis [45].

In order to elucidate the mechanisms explaining the decrease in proliferation observed in TASK-3-depleted cells, cell cycle regulators and apoptosis were evaluated. No significant differences in the expression levels of different cell cycle regulators were detected, which is in contrast to the report by Zuniga et al. [36]. In this work, a significant increase in the expression of the CDK inhibitors p21 and p27 was reported [36]. This disagreement could be explained by differences in the cell lines used, implying that TASK-3 is modulated in a cell type-specific manner [46,47]. In contrast, apoptosis was induced in gastric cancer cells in response to reduced expression of TASK-3 (Figure 4D-G). Therefore, our results indicate that a cytotoxic process is the main cause of the decrease in cell proliferation observed in TASK-3-depleted gastric cancer cells.

Our findings are in agreement with other studies in which the effect of TASK-3 depletion was assessed in other types of cancer cells [37,38,45,48]. More recently, TASK-3 has been involved in the regulation of apoptosis, in part because of its predominantly mitochondrial localization in some cells [49]. Thus, overexpression of TASK-3 in lung cancer cell lines renders these cells resistant to apoptosis induced by TNF, tumor necrosis factor (a mechanism through which cancer cells can defend themselves from the immune system) [37]. Conversely, the dominant negative form of TASK-3 could reverse that resistance [37]. In line with these findings, TASK-3 inhibitors render ovarian cancer cells more sensitive to apoptosis [28]. Furthermore, Nagy et al. [45] demonstrated that knocking down TASK-3 in melanoma cell lines was associated with an increase in apoptosis [45]. These effects were attributed to the predominant mitochondrial expression of TASK-3 and the fact that apoptosis was the result of the activation of caspase-dependent and independent pathways [45]. So far, however, a gap remains in our understanding of how TASK-3 regulates the orchestration of apoptosis.

In order to explore the effects of TASK-3 reduction beyond proliferation, we also assessed cell migration and invasion. These experiments were possible because 40–60% of cells that had incorporated the shRNA targeting TASK-3 did not undergo apoptosis, probably because of the existence of cell clones with different levels of silencing. Migration plays an important role in various physiological processes, including extravasation of leukocytes from the vasculature [50] and migration of fibroblasts during wound healing processes [51]. Importantly, the acquisition of migratory properties is required during tumor progression and metastasis [52]. Ion channels, specifically K^+^ channels, are thought to play a role in migration in part by promoting hydrodynamic changes in cells [52]. Our results demonstrate that the ability to migrate was significant decreased in gastric adenocarcinoma cells with reduced expression of TASK-3. These results are in agreement with other studies carried out in epithelial cell lines, as well as in breast and glioma cancer cells, subjected to pharmacological or genetic inhibition of different K^+^ channels [52,53]. However, it is worth noting that Lee et al. [54] observed that an up-regulation of TASK-3 in MDA-MB-231 breast cancer cells led to a decrease in their migratory capabilities. Strikingly, our own studies showed that knocking down TASK-3 in MDA-MB-231 cells provoked a decrease in cell proliferation associated with an induction of cell cycle arrest [36], further attesting for the complexity and our lack of understanding of the function of these channels in cancer.

Finally, our results indicate that knocking down TASK-3 leads to a significant decrease in the ability of gastric cancer cells to invade in transwell assays. These results do not agree with those reported by Lee et al. [54]. These authors observed that an up-regulation of TASK-3 reduces the invasive capabilities of cells in vitro. This discrepancy could be explained by differences in cell phenotypes and the different pathways in which TASK-3 is involved.

## 4. Materials and Methods

### 4.1. Cell Culture

The human cell lines KATO-III, MKN-45, and HEK-293T were obtained from the American Type Culture Collection. Human gastric carcinoma cells, MKN-45 and KATO-III, were cultured in RPMI-1640 medium (Thermo Scientific, Waltham, MA, USA) supplemented with gentamicin (25 μg/mL), amphotericin B (250 ng/mL), and 10% *v*/*v* of fetal bovine serum (FBS) (Thermo Scientific, Waltham, MA, USA). HEK293-T cells, used for the generation of retroviral particles, were cultured in DMEM high-glucose medium (Thermo Scientific, Waltham, MA, USA) supplemented with gentamicin (25 μg/mL), amphotericin B (250 ng/mL), prophylactic plasmocin (InvivoGen, San Diego, CA, USA), and 10% *v*/*v* of FBS. Cell cultures were kept at 37 °C, 95% humidity, and 5% CO_2_. Cells were cultured until reaching a confluence of approximately 80%. For the maintenance of the culture, periodic passages were made in culture plates (90 × 20 mm).

### 4.2. RNA Isolation and Quantitative RT-qPCR

For total RNA isolation, the TRIzol^®^ reagent (Invitrogen Life Technologies, Carlsbad, CA, USA) was used, essentially following the manufacturer’s protocol. RT-PCR reactions to obtain cDNAs were carried out using the First Strand cDNA Synthesis kit (Thermo Scientific, Waltham, MA, USA), oligo primers (dT), and 1 μg of RNA. The reaction was incubated at 42 °C for 60 min. Quantitative PCR (qPCR) was performed using specific primers (Supplementary Table S1) and the KAPA SYBR^®^ FAST kit (Sigma Aldrich, San Luis, MO, USA). The following genes were analyzed: *TASK-3, TASK-1, CCNA1, CCND1, CCNE1, CDK4, CDKN1A, CDKN1B,* and *RPL19* (see Table S1). For analyses of expression of cell cycle regulators, KATO III and MKN-45 cells were allowed to complete 48 h in culture before being harvested for RNA isolation. The following cycling conditions were used: one cycle at 95 °C for 10 min, followed by 40 cycles at 95 °C for 15 s, 60 °C for 15 s, 72 °C for 20 s, and a final cycle at 95 °C for 1 min in a Stratagene Mx3000P real-time thermal cycler. The results were analyzed with the MxPro Qpcr software (Agilent Technologies, Santa Clara, CA, USA). The relative expression of mRNAs was calculated by the 2^−ΔΔ*C*t^ method. *RPL19* was used to normalize gene expression levels.

### 4.3. Protein Extraction and Western Blotting

Protein extraction was carried out from cell cultures with ≥70% of confluence. Cells were lysed in RIPA buffer containing protease and phosphatase inhibitors. Protein concentration was measured using a protein kit (Bradford Method, 110306, MERCK, Kenilworth, NJ, USA). For western blotting, 60 µg of protein was separated by SDS-PAGE and transferred onto a nitrocellulose membrane. Subsequently, membranes were blocked in PBST buffer (1X PBS and 0.1% Tween-20), containing 5% of nonfat milk, overnight at 4 °C. Finally, blots were incubated with primary antibodies specific for TASK-1 (sc-32067, Santa Cruz Biotechnology, Dallas, TX, USA), TASK-3 (sc-11317, Santa Cruz Biotechnology, Dallas, TX, USA), and GAPDH (sc-365062, Santa Cruz Biotechnology, Dallas, TX, USA) at room temperature for 4 h. After extensive washings with PBST buffer, the membranes were probed with Horseadish Peroxidase Protein (HRP)-conjugated secondary antibodies at room temperature for 2 h. Finally, the Pierce^TM^ ECL western blotting substrate kit (Thermo Scientific, Waltham, MA, USA) was used to detect proteins labeled with HRP, according to the manufacturer’s instructions. To visualize bands, the Omega Lum^TM^ photodocument (Aplegen, San Francisco, CA, USA) was used. The semiquantitative expression of the proteins was determined by means of densitometric analyses using the UltraQuant ID Gel Analysis program (version 5.17.18, San Francisco, CA, USA). Analyses were performed for three independent experiments.

### 4.4. Generation of Retroviral Particles

Retroviral vectors were generated in order to express a short hairpin RNA (shRNA) against the GFP (control) and TASK-3 genes in target cells. Oligo-deoxyribonucleotide sequences (shBP9; sense: 5′-CCG GGC TTC ATC ACG TTG ACT AC-3′; and antisense: 5′-AAT TCA AAA AGC TTC-ATC ACG TTG ACT AC-3′) were first annealed and then subcloned into the vector pMKO.1 puro (Addgene), which carries a resistance cassette that provides resistance to puromycin. Retroviral particles were generated by transfecting pMKO.1-based constructs and packaging plasmids into HEK-293T cells using Lipofectamine 2000 (Invitrogen Life Technologies, San Diego, CA, USA) according to the manufacturer’s instructions. After three days, the medium containing viral particles was collected and stored at –80 °C.

### 4.5. Retroviral Infection

After reaching a confluence of 60–70%, KATO III and MKN-45 cells were infected with retroviral particles carrying shGFP or shBP9 hairpin sequences. In short, culture medium was replaced by an infection mix containing 3 mL of complete medium, 2 mL of retrovirus-containing medium, and 5 μL of polybrene (Sigma Aldrich, San Luis, MO, USA). After further incubation for 8 h at 37 °C and 5% CO_2_, the infection mix was removed and replaced by fresh complete medium. After three days, the selection process started with 2 μg/mL of puromycin (Thermo Scientific, Waltham, MA, USA). The knockdown efficiency was evaluated by RT-qPCR and western blot analyses.

### 4.6. Proliferation and Cell Viability Assays

Confluent cultures of KATO III and MKN-45 cells under different conditions (WT, shGFP, and shBP9) were first trypsinized. Then, 55 × 10^3^ cells/mL were seeded in six-well plates for each condition and in duplicate. Cell proliferation and cell viability were determined by measuring the number of total and viable cells with 0.4% trypan blue vital stain (Thermo Scientific, Waltham, MA, USA) and counted with a LUNA II^TM^ automated cell counter (Logos Biosystems Inc., South Korea, Asia). Curves for both analyses were determined at 0, 24, 48, 72, and 96 h post-seeding in three independent experiments.

### 4.7. Apoptosis Assay

Apoptosis was detected with the Alexa Fluor^®^ 488 AnnexinV/Dead Cell Apoptosis kit (Thermo Scientific, Waltham, MA, USA) according to the manufacturer’s protocol. In short, 20 × 10^4^ cells/mL were seeded in culture plates (90 × 20 mm) for each condition and in triplicate. After 48 h of incubation, KATO III and MKN-45 cells subjected to different conditions were trypsinized. Suspended cells were then washed twice with cold 1X PBS and counted to obtain 2 × 10^6^ cells/mL in 500 μL of 1X annexin-binding buffer. Then, 100 μL of the cell suspension was taken and incubated with Alexa Fluor 488 and 100 μg/mL of propidium iodide at room temperature for 15 min in the dark. After this incubation time, 400 μL of 1× annexin-binding buffer was added, and the detection and analyses of early and late apoptosis were carried out by flow cytometry using a FACSCalibur instrument and BD FACSDiva software (version 6.1.1; BD Biosciences, San Jose, CA, USA).

### 4.8. Wound Healing Assays

A scratch assay was performed to analyze the cell migration in vitro. KATO III and MKN-45 cells under different conditions (WT, shGFP, and shBP9) were cultured as described above. A total of 1.0 × 10^5^ cells/mL were seeded on 12-well plates in triplicate for each condition and incubated for 24 h or until a monolayer was formed. Once a cell monolayer was generated, a scratch or wound was generated by using a 200 μL micropipette tip. Cells were washed with complete medium in the presence of 0.5% FBS to eliminate cells that became detached and again incubated in complete medium with 0.5% FBS (used to avoid the proliferative effect of serum-containing medium) at 37 °C and 5% CO_2_. Three photographs at 10× magnification along the scratch were taken per well at 0, 3, 6, 12, 24, and 48 h. Then, the percentage of wound closure for each condition was determined using the following equation:

% wound closure = (free area at 0 h – free area at × h)/(free area at 0 h).

Digital images were obtained with AmScope software (version 3.7.13522, Irvine, CA, USA) and then analyzed using ImageJ software (version 1.50i, National Institutes of Health, Bethesda, MD, USA).

### 4.9. Cell Invasion Assay

KATO III and MKN-45 cells were cultured in complete medium until reaching a concentration of approximately 2.5 × 10^5^ cells/mL. To carry out invasion assays, a transwell chamber (BD Biosciences, San Jose, CA, USA) was placed in a 24-well culture plate (Sigma-Aldrich, San Luis, MO, USA), and then 100 μL of a stock solution of 3 mg/mL of Matrigel™ (Corning, Tewksbury, MA, USA) was added to the transwell membrane and incubated at 37 °C for 1 h to allow its gelation. Subsequently, 750 μL of RPMI-1640 medium supplemented with 10% FBS was added in the low compartment of the transwell chamber. KATO III and MKN-45 cells were incubated in serum-free medium and trypsinized. Then, 2.0 × 10^5^ cells/mL were seeded onto the Matrigel layer in the upper compartment with serum-free medium. Finally, cells were incubated at 37 °C and 5% CO_2_ for 24 h. After incubation, the cells were stained with 0.5% crystal violet. The invasion ability was determined by counting the number of penetrating cells that were detectable under a light microscope at 20× magnification. Cells present in ten random fields in each well were counted. Each experiment was done in duplicate.

### 4.10. Statistical Analysis

All statistical analyses were performed using the GraphPad Prism (version 7.0, San Diego, CA, USA). Group differences were calculated with one- or two-way ANOVA with Dunnett’s or Tukey’s post-test analysis, respectively. Significant values were all those with a *p* ≤ 0.05. Results were represented as the mean ± SEM of three independent biological experiments.

## 5. Conclusions

In this work, expression of TASK-3 was corroborated in KATO III and MKN-45 gastric cancer cell lines. The shRNA-mediated knockdown of TASK-3 was effective and specific. Knocking down TASK-3 triggers a decrease in the proliferative capacity of KATO III and MKN-45 cells because of a decrease in cell viability. These results were explained by an increase in the proportion of apoptotic cells. In addition, knocking down TASK-3 provoked an inhibitory effect on the migratory and invasive capabilities of KATO III and MKN-45 cells.

Finally, this work opens up a large number of new investigations regarding the functional analyses of TASK-3 in the context of cancer. In particular, it would be of interest to unveil the signaling pathways or proteins that could be affected by TASK-3 depletion. These new functional links will likely lead to a better understanding of the role of this channel in cell proliferation, apoptosis, cell migration, and invasion. Therefore, the possibility of using TASK-3 as a biomarker that can be targeted with specific drugs in the context of cancer therapy is extremely attractive.

## Supplementary Materials

Supplementary Materials can be found at https://www.mdpi.com/1422-0067/20/23/6077/s1.
