# Supplementary material for: TASK-3 Gene Knockdown Dampens Invasion and Migration and Promotes Apoptosis in KATO III and MKN-45 Human Gastric Adenocarcinoma Cell Lines
Source: Int J Mol Sci. 2019 Dec 2;20(23):6077. doi: 10.3390/ijms20236077 (PMC6928893; doi:10.3390/ijms20236077)
Supplement: Supplementary file 1 [file ijms-20-06077-s001.pdf]

**Table S1.** Primer sets used for RT-qPCR analysis.

| Gene | Common Name | GenBank Accession | Abbreviation | Primer Pair, Sense (5′–3′) | Product Size (bp) |
|---|---|---|---|---|---|
| *KCNK9* | K2P9, TASK-3 | NM_001282534.1 | qHsKCNK9_F1 | 5′-GTC TCA TTT TCC CCC ACC TTT CCA G-3′ | 148 |
| | | | qHsKCNK9_R1 | 5′-GGG TGG GGT GAG AAA TGT AAG GCA-3′ | |
| *KCNK3* | K2P3, TASK-1 | NM_002246.2 | qHnP3_F | 5′-GGT GCT CAT CGG CTT CTT CT-3′ | 199 |
| | | | qHnP3_R | 5′-GAA GCT GAA GGC CAC GTA CT-3′ | |
| *CCNA1* | Cyclin A1 | NM_003914.3 | qHsCCNA1_F | 5′-TGA AAT AAG GCA CAG ACC CAA AGC A-3′ | 89 |
| | | | qHsCCNA1_R | 5′-ACC AGC CAG TCC ACC AGA ATC GT-3′ | |
| *CCND1* | Cyclin D1 | NM_053056.2 | qHsCCND1_F | 5′-GCT CCT GTG CTG CGA AGT GGA A-3′ | 126 |
| | | | qHsCCND1_R | 5′-TTT GAA GTA GGA CAC CGA GGG CG-3′ | |
| *CCNE1* | Cyclin E1 | NM_001238.2 | qHsCCNE1_F | 5′-AAG GTT TCA GGG TAT CAG TGG TGC G-3′ | 191 |
| | | | qHsCCNE1_R | 5′-GGC TTT CTT TGC TCG GGC TTT G-3′ | |
| *CDK4* | CDK4 | NM_000075.3 | qHsCDK4_F | 5′-TCG TGA GGT GGC TTT ACT GAG GCG-3′ | 194 |
| | | | qHsCDK4_R | 5′-TCC TTG ATC GTT TCG GCT GGC A-3′ | |
| *CDKN1A* | p21, Cip1 | NM_001291549.1 | qHsCDKN1A_F | 5′-TGT CCG TCA GAA CCC ATG C-3′ | 139 |
| | | | qHsCDKN1A_R | 5′-AAA GTC GAA GTT CCA TCG CTC-3′ | |
| *CDKN1B* | p27, Kip1 | NM_004064.4 | qHsCDKN1B_F | 5′-GGG TCT GTG TCT TTT GGC TCC GA-3′ | 94 |
| | | | qHsCDKN1B_R | 5′-CCG CCT CTC TCG CAC TCT CAA A-3′ | |
| *RPL19* | L19 | NM_000981 | qHsRPL19_F | 5′-CAT CCG CAA GCC TGT GAC G-3′ | 132 |
| | | | qHsRPL19_R | 5′-TGT GAC CTT CTC TGG CAT TCG-3′ | |

K2P, two-pore domain potassium channels; TASK-1 and TASK-3, TWIK-related acid-sensitive K+ channels 1 and 3; DRAM1, CCNA1, CCND1 and CCNE1, Cyclins A1, D1, and E1; CDK4, Cyclin-Dependent Kinase 4; CDKN1A and CDKN1B, Cyclin-Dependent Kinase Inhibitors 1A and 1B; RPL19, ribosomal protein L19.

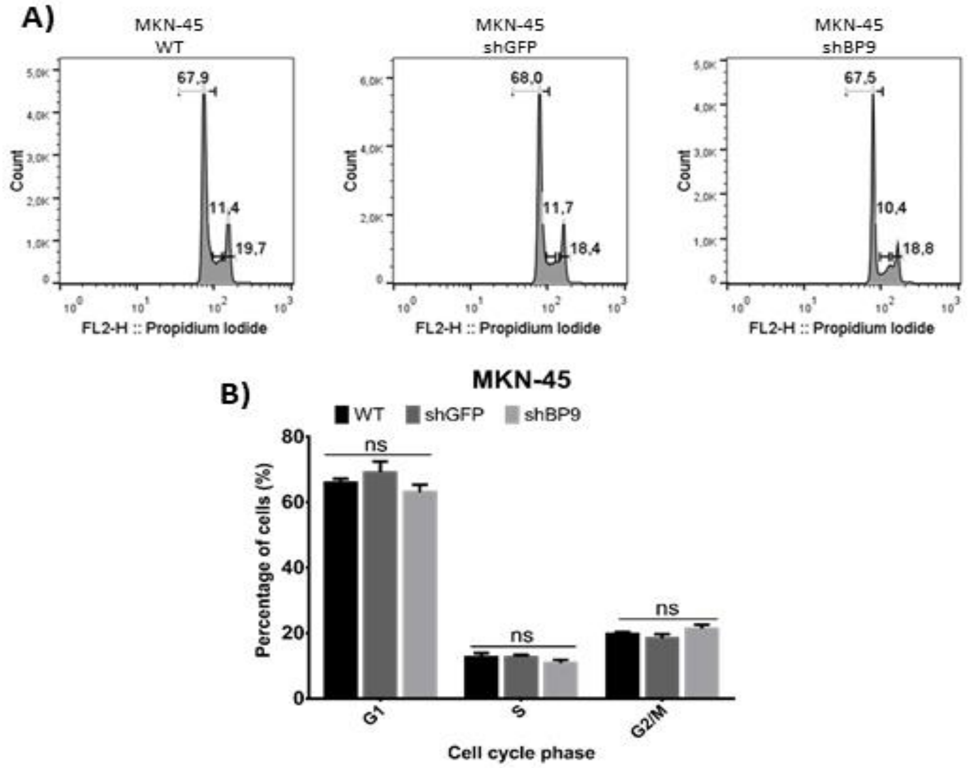


**Figure S1.** Quantutation of cell cyclen phases by propidium iodide staining in MKN-45 cells. (**A**) Histograms. Representing distributions of fluorescence intensity among wild type (WT) MKN-45 cells or the same cells. Transduced with an shRNA against GFP (shGFP) or Task-3 (shBP9) after staining with propidium iodide. (**B**)Percentage of cells in G1, S and G2/M cell cycle phases based on percentages obtained in A. Error bars correspond. To the mean ± SME of three independent experiments. Cell cyle phases were evaluated by two-way ANOVA test with a $p \leq 0.05$ ns = no significant difference.
